# The Survival Relationship between Preoperative Inflammation Markers and Patients with Special Pathological Types of Gastric Cancer

**DOI:** 10.1155/2022/5715898

**Published:** 2022-03-18

**Authors:** Ying Han, Ziyu Zhu, Qi You

**Affiliations:** Department of Gastrointestinal Surgery, Harbin Medical University Cancer Hospital, 150 Haping Road, Nangang District, Harbin 150081, China

## Abstract

**Background:**

The preoperative PLR is closely associated with prognosis of gastric cancer. This aims to research whether the PLR could predict overall survival (OS) of gastric cancer (GC) patients with SRC component.

**Methods:**

The data were collected from Harbin Medical University Cancer Hospital between January 2001 and December 2013 in China. The patients were diagnosed with GC by pathologic examination, which contained SRC component in pathological organization. PLR is obtained from peripheral blood markers (platelets/lymphocytes).

**Results:**

There is a difference in OS between high PLR group and low group, which is verified by Kaplan–Meier analysis and log-rank tests (*P* < 0.001). Moreover, multivariate analysis prove PLR was independent prognostic factor for GC (HR = 1.384, 95% (CI): 1.048–1.828; *P* = 0.022). The preoperative PLR in stage I + II (*P* = 0.033), stage III (*P* < 0.001), SRC component lower than 50% (*P* < 0.001), SRC component equal to or higher than 50% (*P* = 0.044), and R0 resection (*P* < 0.001) GC are still effective.

**Conclusion:**

PLR is a simple, useful, and repeatable predictor of OS in gastric cancer of stages I–III with SRC component and may help clinicians identify patients with high risk and develop a more reasonable follow-up plan.

## 1. Introduction

Worldwide, GC is the fourth most common malignancy, and its mortality rate ranks second [1, 2]. At present, although the technology for treating gastric cancer, such as surgery, chemotherapy, and radiotherapy, has been made progress, the OS of gastric cancer is still poor [3,4].

GC has a relatively rare histopathology type which is signet ring cell (SRC). Studies have shown that GC containing the signet ring cell component has a worse prognosis than other ordinary gastric adenocarcinomas [5, 6]. In addition, the disease incidence of GC has declined on a global scale. Among them, the incidence of intestinal type GC is decreasing, but that of diffuse GC is on the rise. GC containing the signet ring cell component is one of the types of diffuse GC. Moreover, its incidence is also on the rise [7, 8]. The ability to predict the OS of patients is crucial for the selection of treatment options. Similarly, it is greatly valuable to discover the factors, which could accurately evaluate the OS of gastric cancer with SRC.

Systemic inflammatory response indicators include PNI, PLR, and NLR, which are emphasized in the studies of colorectal cancer, GC, and lung malignancy, and have prognostic value. In these studies, preoperative PLR has a great potential prognostic value for the OS of GC [9–11]. Some studies reported the value of PLR for gastric adenocarcinoma [9, 12, 13]. But the function of PLR in predicting OS of GC containing the signet ring cell component is still unclear. Moreover, the GC with SRC is highly malignant and highly morbid. Therefore, this is of great clinical significance to evaluate the function of PLR in predicting OS of GC containing the signet ring cell component.

In order to validate our hypothesis that PLR may better evaluate the OS of GC with SRC components, this study aimed to determine the ability of PLR for predicting postoperative OS of GC containing SRC components.

## 2. Methods

### 2.1. Patients

From January 2001 and December 2013, GC patients who underwent surgery (R0 or R1/R2 resection) were reviewed in Harbin Medical University Cancer Hospital. All patients were diagnosed of gastric cancer by pathologic examination, which contained the SRC component in pathological organization. Exclusion criteria: (1) second primary tumor, (2) received neoadjuvant chemotherapy or radiotherapy, (3) follow-up data and clinicopathological was incomplete, (4) with acute coronary syndromes and inflammatory diseases for almost 1 month.

### 2.2. Data

We collected data contain clinicopathological features, laboratory examinations, and survival duration. Following clinicopathological features, data were collected: TNM, sex, age, pathologic type, tumor diameter and location, and so forth.

Laboratory examinations conducted within one week before surgery were tested for platelet count, WBC count, neutrophil count, hemoglobin count, fibrinogen count, lymphocyte count, albumin count, and globulin count.

PLR = *P*/*L* and NLR = *N*/*L* (*L* = lymphocyte, *N* = neutrophil, *P* = platelet); PNI = albumin concentration + 5 × absolute lymphocyte count [9–11]. Patients were divided into pure SRCC and mixed SRCC according to intracytoplasmic mucin >50% or 10%–50% in tumor. [14].

### 2.3. Follow-Up

The prognosis was collected by regular telephone. The date of surgery is the starting point, and last follow-up or death is the end point. Follow-up time is between August 2001 and November 2018.

### 2.4. Statistical Analysis

This study was analyzed by SPSS 21.0 software. The cut-off values of PLR, PNI, WBC, NLR, hemoglobin, fibrinogen, albumin, and tumor size were selected by ROC, using Chi square test to find out relationships among the PLR and clinicopathological features. Survival analysis was verified by Kaplan–Meier method, and survival rate was compared by log-rank test using the cox proportional hazards regression model to analyze OS of GC patients (*P* < 0.05 was pondered significant for all analyses).

## 3. Results

### 3.1. Optimal Thresholds for Prognostic Factor

As shown in Table 1, the AUC for PLR, WBC, hemoglobin, fibrinogen, albumin, globulin, tumor diameter, PNI, and NLR, were 0.639 (*P* < 0.001), 0.525 (*P* = 0.284), 0.594 (*P* < 0.001), 0.625 (*P* < 0.001), 0.587 (*P* < 0.001), 0.512 (*P* = 0.614), 0.749 (*P* < 0.001), 0.626 (*P* < 0.001) and 0.594 (*P* < 0.001), respectively. The cut-off values were 141.5 for PLR, 3.06 for fibrinogen, 1.95 for NLR, 51.15 for PNI, 41 for albumin, 4.90 for WBC, 122.1 for hemoglobin, 23.3 for globulin, and 5 for tumor diameter by ROC curve analysis.

### 3.2. Patient Characteristics

In Table 2, clinicopathological features were shown. This study enrolled 601 patients, and 391 (65.1%) cases were male and 210 (34.9%) cases were female, respectively. Median age was 55 (22–84). At the end of follow-up, 276 (45.9%) were alive and 325 (54.1%) patients were dead.

### 3.3. PLR and Clinicopathological Features

The factors between PLR and GC patients, such as sex, tumor diameter and location, R0/R1 (R2) resection, WBC (white blood cell), hemoglobin, fibrinogen, albumin, TNM, PNI, and NLR show significant relationship (all *P* < 0.05) (Table 2).

### 3.4. PLR and OS

**Figure 1:**
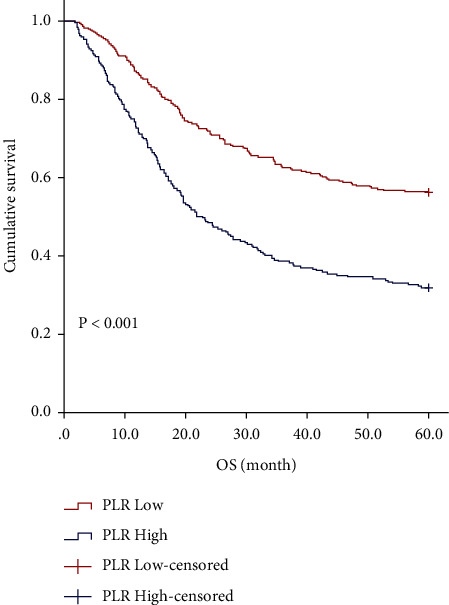
Kaplan–Meier analysis of OS for the PLR of all patients with gastric cancer.

**Figure 2:**
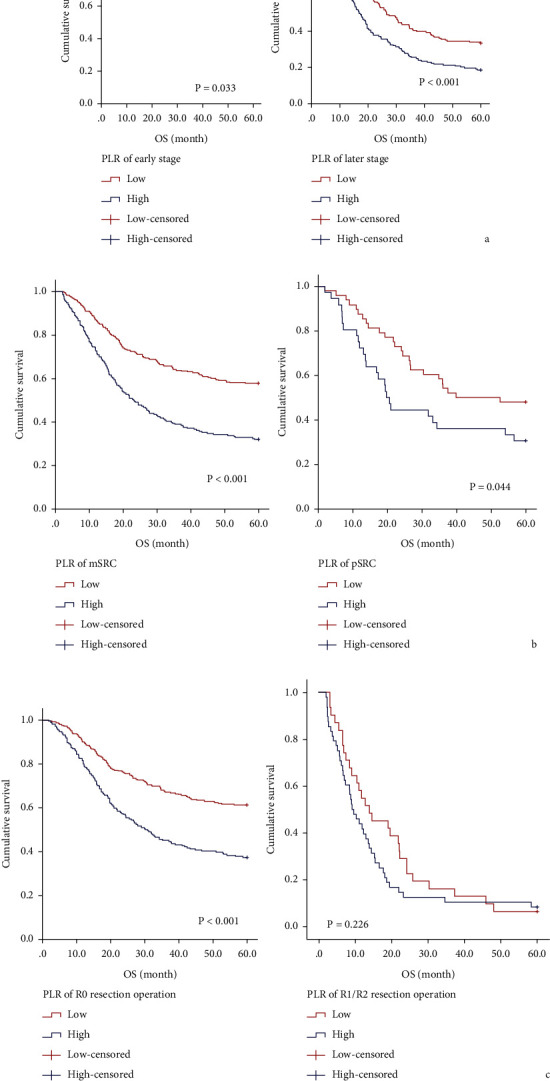
Kaplan–Meier analysis of OS for the PLR of all patients with gastric cancer with related characteristics. (a) pTNM stage: I + II stage and III stage. (b) mSRC or pSRC. (c) R0 or R1/R2 resection.

In Table 3, the five-year survival rates were 56.2% and 31.7% in low and high PLR group, respectively (Figure 1; *P* < 0.001). In addition, PLR in stage I + II (*P* = 0.033; Figure 2(a)1), stage III (*P* < 0.001; Figure 2(a)2), mSRCC (*P* < 0.001; Figure 2(b)1), pSRCC (*P* = 0.044; Figure 2(b)2), and R0 resection (*P* < 0.001; Figure 2(c)1) were still valuable. However, PLR in R1/R2 resection is invalid (*P* = 0.226; Figure 2(c)2).

### 3.5. Univariate and Multivariate Analysis for OS

In Table 4, the PLR, age, tumor location, tumor diameter, R0 or R1/R2 resection, T, N, TNM stage, WBC, NLR, hemoglobin, PNI, fibrinogen, and albumin were statistically significant in univariate analysis (all *P* < 0.05). To further study the significant prognostic factors, this result showed that R0 or R1/R2 resection (2.087, 1.568–2.779, *P* < 0.001), T stage (1.842, 1.488–2.280, *P* < 0.001), N stage (1.366, 1.209–1.543, *P* < 0.001), and PLR (1.384, 1.048–1.828, *P* = 0.022) were still valid in multivariate analysis. However, age, tumor diameter, tumor location, WBC, hemoglobin, fibrinogen, albumin, PNI, and NLR were invalid.

## 4. Discussion

Nowadays, gastric cancer is decreasing on a global scale, but diffuse GC in Lauren classification is still rising. The subtypes of SRC and pSRC in diffuse gastric cancer are also on the rise, which deserves clinical attention [9, 14]. Inflammatory markers such as PLR can be detected in routine laboratory tests before treatment. Thus, inflammatory marker is a simple, cheap, and convenient blood predictor.

Our study finds that PLR could independently predict OS of GC. Cancer and tumor-promoting inflammations are linked [15]. Systemic inflammatory responses act as an important facilitating role in cancer, including initiation, progression, malignant conversion, and metastasis and development [16, 17].

A growing number of research studies certificated that low PLR was link to well OS of many cancers such as breast, liver, esophageal, colon, and gastric cancer [18–20].

There are some relatively popular mechanisms. First of all, necessary condition for the recruitment of granulocytes was platelet-derived signals, which could promote the conformation of early metastatic niches for tumor cells [21]. Second, the bone remodeling alterations before metastasis and the correspondence between primary tumor cells could be promoted by platelets [22]. Third, tumor cells could not be eliminated by immune system with platelet's protection [22, 23]. Platelets could serve as a reservoir that secreted a vast variety of growth factors, which could further increase angiogenesis, tumor growth, and metastasis [24–27]. Moreover, lymphocytes played a main character in tumor-associated inflammatory response [28]. Antitumor activity of lymphocytes was brought by inhibiting tumor proliferation and inducing cytotoxic cell death [29].

At present, more and more scholars study the relationship between PLR and cancer. A research of 26 studies (including 13,964 patients) showed that PLR may be a significant biomarker in the prognosis for various cancers, including GC [30]. The preoperative PLR is a useful and simple predictor in the clinical T2-4GC who underwent curative gastrectomy [31]. A study has indicated that the patients with high PLR level had worse survival condition and nutritional status [32].

Our study confirmed that the PLR was linked to OS (*P* < 0.001). Compared to high PLR patients, those with low PLR would live longer. The value of PLR in GC patients with SRC was further certificated by COX analysis.

While other prognostic factors available in the laboratory were linked to OS by the first step analysis, they did not have independent prognostic value after entering multivariate analysis. The TNM stage was a recognized marker for predicting the OS of GC [33]. Moreover, the function of PLR to predict prognosis survival in stage I + II and III was still effective. This could be an effective complement to the TNM staging's capabilities and scope. In patients with mSRCC and pSRCC, PLR had a predictive ability on OS. In R0/R1 (R2) resection, PLR was valid in patients with R0 resection, but there was no statistical significance in the R1/R2 resection group. Therefore, the above results supported the prognostic value of PLR in I-III GC with SRC component.

At present, *Helicobacter pylori* (*H. pylori*) has been identified as a carcinogen of gastric cancer [34]. Remarkably, one out of every two people is infected with *Helicobacter pylori* worldwide [35]. Similarly, cancers caused by *Helicobacter pylori* infection have a higher incidence compared to other various cancers [36]. Therefore, it is very important to study the relationship between *Helicobacter pylori* and prognostic factors of gastric cancer.

Recently, a study from the Kurdistan region of Iraq showed that patients with a high body mass index had a high failure rate in *Helicobacter pylori* eradication therapy [37]. In addition, Masoodi study indicated that, compared to younger group, *H. pylori* eradication failure rates appear to be higher in older adults. Then, in the elderly group, the eradication failure rate was significantly higher in men than in women [38]. Moreover, recent studies have shown that continued smoking and increased dose of smoking during *Helicobacter pylori* treatment can lead to an increased failure risk of *Helicobacter pylori* eradication. And, there is a higher failure rate in smokers than in nonsmokers during the *Helicobacter pylori* treatment [39]. The incidence of gastric cancer is a cumulative process, and the incidence is relatively higher in the elderly. In addition, the incidence of gastric cancer is higher in men than in women. Furthermore, smoking is also an important factor leading to gastric cancer [40–42]. These factors are not conducive to the treatment of *Helicobacter pylori*. Similarly, a large number of studies have shown that clinicopathological factors such as age, gender, and BMI are risk factors that affect the prognosis of gastric cancer patients [43–46]. Therefore, the research on the therapeutic effect, treatment mode, and drug resistance of *Helicobacter pylori* in gastric cancer patients has very important value and clinical significance.

Recently, the positive effects of *Allium* extracts as an additional treatment for several gastrointestinal cancers also have been widely concerned [47]. A review article showed 7 studies suggested that a high intake of *Allium* vegetable had a relatively beneficial effect for GC. 14 researches on garlic and more than 80% research on onion showed a favourable role of these *Allium* types in gastric cancer [48]. These *Allium* extracts inhibit cancer by multiple mechanisms, including modulating metabolism of carcinogen, inhibiting the formation of carcinogens, inhibiting angiogenesis, enhancing immune system, inhibiting cell proliferation and increasing apoptosis, and inhibiting mutagenesis and genotoxicity [49]. In vivo and in vitro studies showed that *Allium* extract can prevent and inhibit the progression of carcinogenesis in gastrointestinal tumors [47]. Ongoing research on *Helicobacter pylori* and *Allium* extracts is extremely valuable, which is also the direction we will explore in the next step.

There were several main limitations in this study. First, our patients lacked disease-free survival or recurrence-free survival. Despite lack of these data, OS was an internationally recognized standard for cancer prognosis, [50] and a subgroup analysis was added to our study to complement the findings of this study. Second, our data were from a single-center. However, the treatment of our patients, the collection of clinical pathological features, and follow-up were based on uniform criteria. And, the data in this study was a large number of consecutive samples, providing an effective basis for investigating the prognostic ability of PLR for evaluating GC patients with SRC.

## 5. Conclusions

PLR can predict OS in gastric cancer patients with SRC component, which has a special value. This factor might be a supplement to the TNM stage system and better be able to help clinicians identify patients with high risk and develop a more reasonable follow-up plan.

## Figures and Tables

**Table 1:** The optimal thresholds for prognostic factor by the ROC curves.

Prognostic score	Area under the ROC curve (95%CI)	*P* value
PLR	0.639 (0.595–0.683)	<0.001
WBC	0.525 (0.479–0.572)	0.284
Hemoglobin	0.594 (0.548–0.639)	<0.001
Fibrinogen	0.625 (0.580–0.670)	<0.001
Albumin	0.587 (0.542–0.633)	<0.001
Globulin	0.512 (0.466–0.558)	0.614
Tumor size	0.749 (0.710–0.789)	<0.001
PNI	0.626 (0.582–0.671)	<0.001
NLR	0.594 (0.549–0.639)	<0.001

**Table 2:** The clinicopathological characteristics of 601 patients with gastric cancer.

Variables	PLR ＜ 141.5 (cases)	PLR ≥ 141.5 (cases)	*X* ^*2*^ value	*P* value
Total	349	252		
Sex
Male	243	148	7.644	0.006
Female	106	104		
Age (years)			0.272	0.602
<55	164	113		
≥55	185	139		
SRC content (%)
<50	301	216	0.034	0.853
≥50	48	36		
Tumor diameter (cm)			27.457	<0.001
<5	168	68		
≥5	181	184		
R0/R1 (R2) resection			13.244	<0.001
R0	318	204		
R1/R2	31	48		
pT			28.946	<0.001
*T*_1_	68	16		
*T*_2_	47	20		
*T*_3_	36	35		
*T*_4_	198	181		
pN			24.968	<0.001
N_0_	116	58		
N_1_	61	33		
N_2_	76	45		
N_3_a	64	65		
N_3_b	32	51		
pTNM			27.881	<0.001
I + II	164	65		
III	185	187		
Tumor location			13.461	0.019
Lower stomach	228	141		
Middle stomach	47	43		
Upper stomach	25	15		
LM stomach	35	31		
MU stomach	2	10		
LMU stomach	12	12		
WBC			20.707	<0.001
<4.90	75	97		
≥4.90	274	155		
Hemoglobin			75.177	<0.001
<122.1	58	125		
≥122.1	291	127		
Fibrinogen			27.182	<0.001
<3.06	204	93		
≥3.06	145	159		
Albumin			8.327	0.004
<41	140	131		
≥41	209	121		
Globulin			2.406	0.121
<23.3	117	100		
≥23.3	232	152		
PNI			65.098	<0.001
<51.15	133	180		
≥51.15	216	72		
NLR			111.182	<0.001
<1.95	259	78		
≥1.95	90	174		

**Table 3:** Association of PLR with OS by Kaplan–Meier analysis.

Variables	Patients (case (%))	Mean OS with 95% CI (months)	*P* value
Sex			0.840
Male	391 (65)	38.3 (36.1–40.5)	
Female	210 (35)	37.6 (34.6–40.7)	
Age (years)			0.001
<55	277 (46.1)	41.4 (38.9–44.0)	
≥55	324 (53.9)	35.2 (32.7–37.6)	
SRC content			0.371
<50	517 (86.0)	38.3 (36.4–40.3)	
≥50	84 (14.0)	36.5 (31.8–41.1)	
Tumor diameter (cm)			<0.001
<5	236 (39.3)	49.2 (46.9–51.6)	
≥5	365 (60.7)	30.8 (28.6–33.1)	
R0/R1 (R2) resection			<0.001
R0	522 (86.9)	41.3 (39.4–43.1)	
R1/R2	79 (13.1)	16.9 (13.3–20.5)	
pT			<0.001
*T*_1_	84 (14.0)	59.4 (58.4–60.3)	
*T*_2_	67 (11.1)	53.2 (49.6–56.8)	
*T*_3_	71 (11.8)	39.1 (34.2–44.0)	
*T*_4_	379 (63.1)	30.5 (28.3–32.6)	
pN			<0.001
N_0_	174 (30.0)	52.7 (50.3–55.1)	
N_1_	94 (15.6)	44.3 (40.4–48.2)	
N_2_	121 (20.1)	36.9 (33.2–40.7)	
N_3_a	129 (21.5)	27.0 (23.3–30.7)	
N_3_b	83 (13.8)	19.1 (15.5–22.7)	
pTNM			<0.001
I + II	229 (38.1)	52.6 (50.6–54.6)	
III	372 (61.9)	29.1 (26.9–31.2)	
Tumor location			<0.001
Lower stomach	369 (61.4)	41.4 (39.2–43.6)	
Middle stomach	90 (15.0)	35.9 (31.2–40.6)	
Upper stomach	40 (6.7)	31.6 (24.4–38.8)	
LM stomach	66 (11.0)	32.8 (27.5–38.2)	
MU stomach	12 (2.0)	27.9 (14.8–40.9)	
LMU stomach	24 (3.9)	24.3 (16.4–32.3)	
WBC			0.041
<4.90	172 (28.6)	35.3 (31.9–38.6)	
≥4.90	429 (71.4)	39.2 (37.1–41.3)	
Hemoglobin			<0.001
<122.1	183 (30.4)	31.4 (28.2–34.6)	
≥122.1	418 (69.6)	41.0 (38.9–43.1)	
Fibrinogen			<0.001
<3.06	297 (49.4)	42.6 (40.1–45.0)	
≥3.06	304 (50.6)	33.7 (31.2–36.1)	
Albumin			<0.001
<41	271 (45.1)	33.5 (30.8–36.2)	
≥41	330 (54.9)	41.8 (39.5–44.1)	
Globulin			0.098
<23.3	217 (36.1)	36.0 (33.0–39.1)	
≥23.3	384 (63.9)	39.2 (37.0–41.4)	
PNI			<0.001
<51.15	313 (52.1)	32.4 (29.9–34.9)	
≥51.15	288 (47.9)	44.2 (41.8–46.6)	
NLR			<0.001
<1.95	337 (56.1)	42.2 (39.9–44.4)	
≥1.95	288 (47.9)	32.8 (30.1–35.5)	
PLR			<0.001
<141.5	349 (58.1)	43.0 (40.8–45.2)	
≥141.5	252 (41.9)	31.2 (28.4–34.0)	

**Table 4:** Univariate and multivariate cox regression analysis for OS.

Variables	Univariate analysis		Multivariate analysis	
	HR (95%CI)	*P* value	HR (95%CI)	*P* value
Sex	1.024 (0.815–1.286)	0.840	—	—
Age	1.474 (1.181–1.841)	0.001	1.153 (0.914–1.455)	0.231
SRC content	0.872 (0.645–1.178)	0.372	—	—
Tumor diameter	3.485 (2.672–4.547)	<0.001	1.340 (0.995–1.804)	0.054
Tumor location	1.229 (1.146–1.317)	<0.001	1.011 (0.940–1.088)	0.762
R0/R1 (R2) resection	4.318 (3.309–5.635)	<0.001	2.087 (1.568–2.779)	<0.001
pT	2.471 (2.074–2.944)	<0.001	1.842 (1.488–2.280)	<0.001
pN	1.759 (1.618–1.912)	<0.001	1.366 (1.209–1.543)	<0.001
pTNM	5.643 (4.166–7.642)	<0.001	0.971 (0.609–1.550)	0.903
WBC	0.784 (0.621–0.991)	0.041	0.914 (0.698–1.195)	0.510
Hemoglobin	0.573 (0.458–0.718)	<0.001	1.085 (0.841–1.399)	0.530
Fibrinogen	1.771 (1.418–2.213)	<0.001	1.001 (0.780–1.285)	0.993
Albumin	0.623 (0.501–0.775)	<0.001	1.067 (0.786–1.448)	0.678
Globulin	0.828 (0.663–1.036)	0.099	—	—
PLR	2.047 (1.645–2.547)	<0.001	1.384 (1.048–1.828)	0.022
PNI	0.501 (0.400–0.628)	<0.001	0.781 (0.554–1.100)	0.157
NLR	1.691 (1.360–2.102)	<0.001	0.986 (0.755–1.287)	0.918

## Data Availability

Due to the privacy and confidentiality of the clinical data of this center, data sharing is not applicable.
